# Application of the PHENotype SIMulator for rapid identification of potential candidates in effective COVID-19 drug repurposing

**DOI:** 10.1016/j.heliyon.2023.e14115

**Published:** 2023-03-06

**Authors:** Naomi I. Maria, Rosaria Valentina Rapicavoli, Salvatore Alaimo, Evelyne Bischof, Alessia Stasuzzo, Jantine A.C. Broek, Alfredo Pulvirenti, Bud Mishra, Ashley J. Duits, Alfredo Ferro, RxCOVEA Framework

**Affiliations:** a Department of Computer Science, Mathematics, Engineering and Cell Biology, Courant Institute, Tandon and School of Medicine, New York University, New York, USA; b Institute of Molecular Medicine, The Feinstein Institutes for Medical Research, Northwell Health, Manhasset, NY, USA; c Department of Medicine, Donald and Barbara Zucker School of Medicine at Hofstra, Northwell Health, Manhasset, NY, USA; d Red Cross Blood Bank Foundation Curaçao, Willemstad, Curaçao; e Department of Medical Microbiology and Immunology, St. Antonius Ziekenhuis, Niewegein, the Netherlands; f Department of Physics and Astronomy, University of Catania, Italy; g Bioinformatics Unit, Department of Clinical and Experimental Medicine, University of Catania, Italy; h Department of Advanced Biomedical Sciences, University of Naples Federico II, Via Pansini, Naples, Italy; i School of Clinical Medicine, Shanghai University of Medicine and Health Sciences, Pudong, Shanghai, China; j Insilico Medicine, Hong Kong Special Administrative Region, China; k Department of Chemical Sciences, University of Catania, Italy; l Simon Center for Quantitative Biology, Cold Spring Harbor Lab, Long Island, USA; m Curaçao Biomedical Health Research Institute, Willemstad, Curaçao; n Institute for Medical Education, University Medical Center Groningen, Groningen, the Netherlands

**Keywords:** COVID-19, Drug repurposing, Systems biology, Cellular simulation models, Cellular SARS-CoV-2 signatures, Cellular host-immune response, PHENSIM, PHENotype SIMulator, SARS-CoV-2, Severe acute respiratory syndrome coronavirus 2, COVID-19, Coronavirus disease 2019, DEGs, Differentially Expressed Genes, ACE2, Angiotensin-converting enzyme 2, IFN, Interferon, NHBE, Normal human bronchial epithelial cells, MOI, Multiplicity of infection, ISGs, IFN-stimulated genes, MITHrIL, Mirna enrIched paTHway Impact anaLysis, TLR, Toll-like Receptor, Calu-3, Epithelial cell line, Caco-2, Human colon epithelial carcinoma cell line, DEPs, Differentially expressed proteins, HCQ-CQ, (Hydroxy)chloroquine, 2DG, 2-Deoxy-Glucose, MP, Methylprednisolone

## Abstract

The current, rapidly diversifying pandemic has accelerated the need for efficient and effective identification of potential drug candidates for COVID-19. Knowledge on host-immune response to SARS-CoV-2 infection, however, remains limited with few drugs approved to date. Viable strategies and tools are rapidly arising to address this, especially with repurposing of existing drugs offering significant promise. Here we introduce a systems biology tool, the PHENotype SIMulator, which -by leveraging available transcriptomic and proteomic databases-allows modeling of SARS-CoV-2 infection in host cells *in silico* to *i)* determine with high sensitivity and specificity (both>96%) the viral effects on cellular host-immune response, resulting in specific cellular SARS-CoV-2 signatures and *ii)* utilize these cell-specific signatures to identify promising repurposable therapeutics. Powered by this tool, coupled with domain expertise, we identify several potential COVID-19 drugs including methylprednisolone and metformin, and further discern key cellular SARS-CoV-2-affected pathways as potential druggable targets in COVID-19 pathogenesis.

## Graphical abstract

**Figure:**
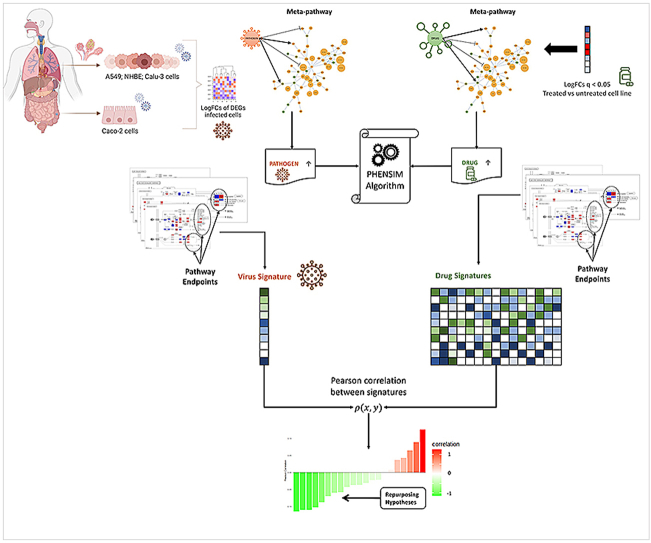
**Application of the PHENotype SIMulator:** By modeling human host-cell infection with a pathogen in silico - in this case SARS-CoV-2 - we can acquire a cell-specific *viral signature* and formulate multiple drug repurposing hypotheses; (I) logFold Changes (logFCs) of Differentially Expressed Genes (DEGs) arising from transcriptomic genome wide expression analysis of infected vs. baseline uninfected cells are used to represent a virus in the meta-pathway; (II) we run the PHENSIM simulation by upregulating the viral node and collect all perturbation values computed by PHENSIM for pathway endpoints to define a cell-specific *pathogen signature*. (III) The same process is applied to expression data arising from whole transcriptome-wide expression analysis of drug treated vs. mock-treated cell lines, yielding a cell-specific drug signature. This process is iterated for each drug we wish to test and collected in a database of drug signatures. (IV) Finally, a Pearson correlation analysis between the pathogen and each drug signature is utilized to score repurposing candidates.

## 1 Introduction

The rapid emergence and spread of the virulent novel severe acute respiratory syndrome coronavirus 2 (SARS-CoV-2) has hijacked and largely disrupted human civilization as we know it, bringing about countless global challenges but also the urgent need for innovative vaccine and drug discovery [1]. Soon after its emergence in Wuhan (Eastern China) in late 2019, coronavirus disease 2019 (COVID-19) was declared a pandemic by the World Health Organization [2], as it continuously spreads and holds the world hostage. As of May 2022, over 513 million COVID-19 cases and 6.2 million deaths have been reported worldwide. It is apparent that our civilized, well-organized and hitherto functioning societies were not adequately prepared nor equipped to deal with the high infectivity, transmissibility, mortality and global impact of the COVID-19 pandemic. While vaccine development and deployment are well underway, widespread global distribution remains challenging, as is the waning vaccine protective effectivity against emerging mutated virus variants [3]. At present only an antiviral (Remdesivir) and glucocorticoids (Dexamethasone/Methylprednisolone) have been approved for treatment of severe COVID-19. Recently, for COVID-19 patients with high risk for developing severe disease, two oral antiviral drugs (Paxlovid and Molnupiravir) [4,5] received emergency use authorization. Approved virus-neutralizing antibody cocktails that received emergency use authorization for treatment of mild to moderate COVID-19 in high-risk patients [6] have been proven to be less effective against the current Omicron variant [7]. Otherwise, no established drug is available to prevent or adequately treat COVID-19 and in the absence of a clear etiological understanding, treatment has remained largely supportive and symptomatic [8,9].

Therefore, next to *in vitro* studies, *in silico* studies are of great value for rapid and effective drug discovery. Indeed, computational structure-based drug design and immuno-informatics have recently resulted in identification of potential SARS-CoV-2 target proteins and drugs that are being selected for further testing [8,10,11]. Another promising avenue for obtaining effective and readily available therapeutic strategies is the repurposing of drugs already approved for other indications. Drug repurposing strategies provide an attractive and effective approach based on available drug characteristics – drug-related pharmacology and toxicology – for rapid therapeutic selection [12]. If we could, with higher probability, identify and pre-select the most promising hypothesis-based candidates using *in silico* systems biology tools, prior to costly and laborious *in vitro* and *in vivo* experiments and ensuing clinical trials, we could significantly improve disease-specific drug development [13].

Several *in silico* techniques have been developed, mainly making use of molecular modeling of key viral proteins for virtual screening of drug candidates simulating receptor-drug molecular dynamics [9,10]. In order to increase the effectivity of identifying candidate drugs for combating COVID-19, it is crucial to build on a more in-depth knowledge of the molecular basis of the immune signaling pathways regarding host-virus interaction and SARS-CoV-2-induced immunopathology. Only if we better understand how this particular virus affects host cells in detail, on a transcriptomic, proteomic level and beyond [8,9,14], will we be able to effectively treat COVID-19 patients. It is becoming evident that treatment should not only focus on direct anti-viral effects in mild cases but should also encompass potential (cytokine storm induced) aberrant host-response in severe cases [8,15,16]. Taken together, this points towards the importance of a more detailed and targeted approach for COVID-19, where antivirals or steroids alone might not suffice and specifically targeting the (aberrant) host-response is imperative [8,11,12].

Recently in literature, tools and algorithms devised to perform simulation on biological networks have been described [17,18]. Here we aim to utilize our systems biology tool, the *PHEN*otype *SIM*ulator (PHENSIM) [19], to leverage the power of pathway analysis by simulating tissue-specific infection of host cells of SARS-CoV-2 and subsequently perform *in silico* drug selection for potential repurposing. PHENSIM is a web-based user-friendly computational tool that allows phenotype prediction on selected cells, cell-lines and tissues, using a probabilistic algorithm [20] via “message passing” [21] across a network of meta-pathways [22]. These meta-pathways are obtained by joining all validated biological pathways, enriched with gene regulatory elements [23]. The algorithm thus computes, under-specified biological contexts, by iteratively propagating the effects and alterations of one or more biomolecules (differentially expressed genes (DEGs), proteins, microRNAs, or metabolites), thus making use of published virus-human interaction data [11]. Here we compare our results with available data from recently published *in vitro* studies based on transcriptomics and proteomics in different model systems [9,14]. Relevant and significantly affected pathways are further detailed on a protein interaction level. Finally, we show the potential of PHENSIM in selecting promising hypothesis-driven COVID-19 drug candidates suggestive of in-vitro anti-viral effects, which has applicability to other diseases and broader aspects of clinical practice, thereby outlining the potential power of PHENSIM in aiding effective drug repurposing in COVID-19 and beyond.

## 2 Results

### 2.1 PHENSIM model: from *in vitro* to *in silico*

Innovative approaches to rapidly elucidate a pathogens’ mechanism of action have proven crucial for containing the global burden of communicable diseases. The PHENSIM approach, described here, is based on the definition of a newly introduced protocol for *in silico* simulation of novel emerging pathogens, such as SARS-CoV-2, and it aims at elucidating distinct host-responses and molecular mechanisms triggered by that particular pathogen, all while defining possible candidate drugs for indication repositioning.

**Fig. 1:**
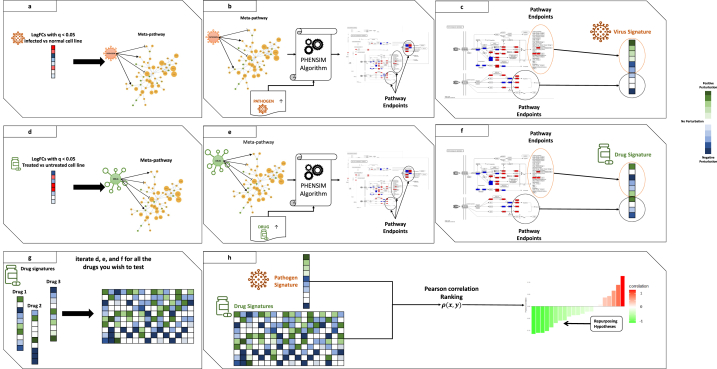
**Schematic representation of the PHENSIM Drug repurposing Strategy**. Outline for our approach to acquire a cell-specific viral signature *in silico* and formulate repurposing hypotheses*:* (a) first, logFold Changes (logFCs) of Differentially Expressed Genes (DEGs) arising from transcriptomic genome wide expression analysis of infected vs. baseline uninfected cells are used to represent a virus in the meta-pathway; (b) then, we run the PHENSIM simulation by upregulating the viral node; (c) therefore, we collect all perturbation values computed by PHENSIM for pathway endpoints to define a cell-specific *pathogen signature*. (d) The same process is applied to expression data arising from whole transcriptome-wide expression analysis of treated vs. mock-treated cell lines, (e) to perform a PHENSIM simulation of the drug activity, (f) yielding a cell-specific drug signature. (g) Thus, this process is iterated for each drug we wish to test and collected in a database of drug signatures. (h) Finally, a Pearson correlation analysis between the pathogen and each drug signature is used to score repurposing candidates, yielding hypothesis for further laboratory tests. In panels (a) and (d), we report upregulated DEGs in red and downregulated ones in blue. In panels (c), (f), and (g), we report positively perturbated endpoints in green and negatively perturbated endpoints in blue. Finally, in panel (h), negative correlation (reported in green) predicts promising drug candidates that inhibit the pathogen signature and positive correlation (reported in red) suggests exacerbation of the viral signature when introducing the drug.

For our strategy to be viable, even when only limited direct knowledge is available on the host-pathogen interaction, we need direct infection (*in vitro*) data that can be exploited to predict such interactions. To acquire this knowledge, we therefore employ transcriptomic and proteomic experiments of *in vitro* infected vs. normal, pathogen-free cell lines. When available, we leverage Differentially Expressed Genes (DEGs) to simulate the direct and indirect effect of the virus on a host without a priori knowledge regarding the mechanism of infection. Using DEGs as input for our cell *PHEN*otype *SIM*ulator PHENSIM [20,24] we define a signature of pathogen predicted effects on human pathways (*pathogen alterations profile*; here termed the “*viral signature*”; see Fig. 1). To build the viral signature, we use pathway endpoints; an endpoint is a biological element in a pathway whose alteration, based on current knowledge, affects the phenotype in a specific way [25].

By leveraging PHENSIM we aimed to determine the impact of such viral infection induced alterations on an array of human cell lines *in silico.* Simulation results are used to define a “viral signature”, which can then be employed to identify candidate drugs. Once a cell-specific SARS-CoV-2 viral signature is defined, potential repositioning drugs can be identified by building a “drug signature” database queried by means of a similarity measure using pathway endpoints (Fig. 1). Given a candidate drug identified through a database (i.e. Drugbank or Pubchem) and literature (Pubmed) search, we define all known targets and alterations (up/down-regulations caused by the drug). Alterations are then provided as input to PHENSIM, together with the corresponding cell-specific *viral signature.* Next, distinct pathways endpoints [25] are identified and resulting *drug signatures* relating to a specific candidate can subsequently be compared with acquired *viral signatures* to evaluate the inhibitory potential of that candidate drug. Both viral and drug signatures are collected in a database, where a similarity search is performed using a Pearson correlation *ρ*(x,y) [26]; see methods section equation (1). All drugs whose correlation with the virus is negative (green) are considered possible repositioning candidates, since they predict inhibition of the viral signature, whereas a positive correlation (red) suggests exacerbation of the viral signature when introducing the candidate drug.

### 2.2 Validation of PHENSIM transcriptomic strategy in SARS-CoV-2-infected host cells

To validate our PHENSIM model on a transcriptomic level in the context of SARS-CoV-2, we sought to replicate the *in vitro* experiments using publicly available data presented by Blanco-Melo et al. [14]. The in-depth transcriptomic analysis of SARS-CoV-2 elicited host-response by Blanco-Melo et al. recently revealed an inappropriate inflammatory response driven by reduced innate antiviral defenses, with low or delayed type I and type III interferon (IFN) and exaggerated inflammatory cytokine response, with elevated chemokines and IL-6 [14].

**Fig. 2:**
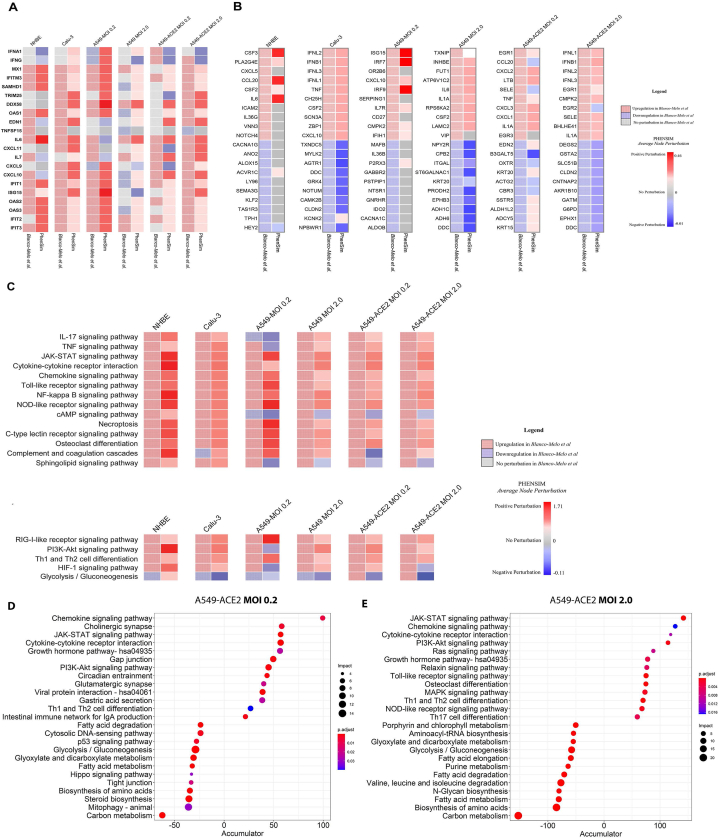
***In silico* PHENSIM prediction of host transcriptional response to SARS-Cov-2.***In vitro* results from *Blanco-Melo* et al. (left column; checkered boxes) are compared to *in silico* PHENSIM predictions (right; solid) for all evaluated respiratory related cells assessed; NHBE, Calu-3, A549 cells at low (0.2) and high (2.0) MOI, ± ACE2 transduction respectively. **A)** Heatmap depicting the perturbation of a select subset of anti-viral, ISGs and inflammatory genes. **B)** Heatmaps depicting unbiased analysis of the top-10 upregulated (red) and top-10 downregulated (blue) DEGs from *Blanco-Melo* et al. (left) with side-by-side PHENSIM predictions (right). For *A&B*, legend shows denoted perturbations for PHENSIM prediction and Blanco-Melo et al. See legend box for DEG annotation. **C)** Heatmap depicts whole genome pathway analysis as predicted by PHENSIM for a select set of signaling pathways of interest in all assessed cell types. Pathway selection was based on highlighted pathways affected by SARS-CoV-2 infection. Color gradient depicts the average pathway perturbation as predicted in our PHENSIM *in silico* experiments. **D&E)** MITHrIL pathway analysis was used to assess top meta-pathways for ***D)*** A549-ACE2 MOI 0.2 (low viral load) and ***E)*** A549-ACE2 MOI 2.0 (high viral load), according to impact (circle size) and significance (color-gradient for adjusted p-value) for the top 12 up- (+accumulator) and down-regulated pathways. The accumulator is the accumulation/sum of all perturbations computed for that particular pathway. NHBE; Normal Human Bronchial Epithelial cells, Calu-3; Cultured human airway epithelial cells, A549; Transformed lung alveolar cells, ACE2; angiotensin-converting enzyme, MOI; multiplicity of infection. DEGs; Differentially expressed genes, ISGs; IFN-stimulated genes.

As SARS-CoV-2 largely affects the lungs and respiratory tract, and because of its apparent affinity for lung tissue, the authors make use of several respiratory epithelial cell lines to assess the transcriptomic host-response. Here we use PHENSIM to reproduce transcriptomic effects *in silico*, as described *in vitro* for the following cell lines, namely undifferentiated normal human bronchial epithelial (NHBE) cells, cultured human airway epithelial cells (Calu-3) cells and A549 lung alveolar cells. The comparison of these results is depicted in Fig. 2. A549 cells are described to be relatively non-permissive to SARS-CoV-2 replication in comparison to Calu-3 cells, which is attributed to low expression of the viral entry receptor angiotensin-converting enzyme (ACE)2 [14,27]. Thus, A549 cells were transduced with human ACE2 (A549-ACE2), which enabled apparent SARS-CoV-2 replication at low-MOI (multiplicity of infection of 0.2). Furthermore, to induce significant IFN-I and –III expression, a high MOI of approximately 2–5 was necessary.

Here we leveraged the data published by Blanco-Melo et al. [14]. To run our PHENSIM simulation pipeline. In Fig. 2A we show representative genes, namely anti-viral, IFN stimulated genes (ISGs) and inflammatory cytokines and chemokines, considered important for the course of SARS-CoV-2 infection. The heatmap shows perturbed expression, either up- or down-regulated, based on results obtained by *in vitro* (left column for each depicted cell-line) experiments for the different cells assessed in comparison to *in silico* PHENSIM predictions (right column; Fig. 2A). An unbiased approach of this predictive comparison is shown in Fig. 2B, displaying the top 10 up- and downregulated DEGs based on *in vitro* SARS-CoV-2 infection, as assessed in the different cells at low and high MOI (0.2 and 2) and with ACE2 addition in A549 lung alveolar cells. For each of the top *in vitro* acquired DEGs (left; checkered boxes), the PHENSIM predicted result is shown side-by-side (right). At first glance, PHENSIM reaches high predictive accuracy for Calu-3 human airway epithelial cells and A549-ACE2 and high MOI of 2, at least for the top DEGs (Fig. 2B). To quantify the overall predictive accuracy of PHENSIM, genome-wide transcriptomic data was assessed for all scenarios as described in Fig. 2. Overall accuracy of *in vitro* predicted transcriptomic results are shown in Table 1, ranging from 51.66 %-for A549-ACE2 MOI 0.2 - to 83.74% for NHBE cells. Sensitivity of perturbation prediction for nodes accurately predicted as perturbed, ranged from 95.83 to 100.00% sensitivity with 97.67–99.86% specificity for this in-depth SARS-CoV-2 transcriptomic analysis. Furthermore, the positive predictive value (PPV) and False negative rate (FNR) are shown for each tested scenario (see Table 1).

**Table 1:** PHENSIM transcriptomic predicted values from *Blanco*-*Melo* et al. 2020.

	Overall Accuracy	Nodes Predicted as perturbed			Nodes predicted as non-perturbed	
		PPV	Sensitivity	Specificity	PPV	FNR
A549-ACE2 MOI 0.2	51.66%	93.50%	96.72%	97.67%	58.90%	41.10%
A549-ACE2 MOI 2	71.72%	96.88%	99.24%	99.13%	60.04%	39.96%
A549 MOI 0.2	83.74%	68.75%	100.00%	99.86%	85.64%	14.36%
A549 MOI 2	78.20%	97.41%	97.20%	99.58%	77.47%	22.53%
Calu-3	77.17%	96.93%	99.34%	99.30%	76.55%	23.45%
NHBE	82.43%	67.65%	95.83%	99.69%	86.48%	13.52%

PPV; Positive predictive value, FNR; False negative rate, NHBE; Normal Human Bronchial Epithelial cells. *Blanco*-*Melo* et al., Cell 2020 [14].

In order to further verify PHENSIM's robustness in whole genome pathway analysis, we next explored PHENSIM's ability to predict significantly affected signaling pathways in SARS-CoV-2 infection. In Fig. 2C we highlight PHENSIM's predicted perturbation of a select set of affected pathways during infection, as recently identified to be of importance by Catanzaro et al. 2020 and Draghici et al. 2020 (also see Supplementary Figs. S1 and S2), such as IL-17, JAK-STAT and TNF signaling pathways, Toll-like Receptor (TLR), NOD-like receptor and RIG-I-like receptor signaling pathways as well as complement and coagulation cascades.

To further evaluate the reliability of our PHENSIM pathway analysis prediction *in silico*, we compared our results with those obtained using our previously described MITHrIL (Mirna enrIched paTHway Impact anaLysis) tool [25] to analyze the Blanco-Melo et al. [14] acquired *in vitro* data (Fig. 2D and E). Given DEGs, MITHrIL first computes a perturbation for each gene in the meta-pathway (as described in Methods section). The perturbation can be considered as the predicted state that the node will have given the input DEGs. Next, we sum the perturbation of all nodes for each pathway to acquire the "accumulated perturbation," or the Accumulator. The accumulator is equivalent to a pathway expression and is a sum of all perturbations computed for that particular pathway. MITHrIL pathway analysis for A549-ACE2 at low viral load (MOI 0.2) revealed Chemokine, JAK-STAT, PI3K-Akt signaling and cytokine-cytokine interaction as a few of the top upregulated pathways, according to impact (circle size), significance (color-gradient for adjusted p-value) and accumulated perturbation computed for that particular pathway (accumulator).

For A549-ACE2 at high viral load (MOI 2.0; Fig. 2E), next to similar pathways at low viral MOI, Toll-like receptor (TLR) and NOD-like receptor signaling were among the top pathways observed, corresponding to the observation that high viral MOI was needed to induce significant type I IFN signaling [14]. Interestingly, both at low and high MOI various metabolic pathways were significantly affected with a negative accumulator. Overall, the MITHrIL analysis results show the most affected pathways to be superimposable onto the PHENSIM *in silico* predicted results.

### 2.3 Modeling proteomics in SARS-CoV-2-infected host cells by leveraging PHENSIM

Using combinatorial profiling of proteomics and translatomics to study host-infection on a cellular and molecular level give opportunity to study relevant viral pathogenicity in the search of potential drug targets [9]. As SARS-CoV-2 has been detected in stool and can replicate in gastrointestinal cells [28,29], Bojkova et al. use the human colon epithelial carcinoma cell line *Caco-2* to study SARS-CoV-2 infection [9]. With their novel method, multiplexed enhanced protein dynamics (mePROD) proteomics, they determined SARS-CoV-2-specific translatome and proteome changes at high temporal resolution [30], and were able to quantify translational changes occurring during SARS-CoV-2 infection *in vitro* over the course of 24 h at multiple timepoints (at 2, 4, 10 and 24h) [9].

#### 2.3.1 PHENSIM proteomic validation

**Fig. 3:**
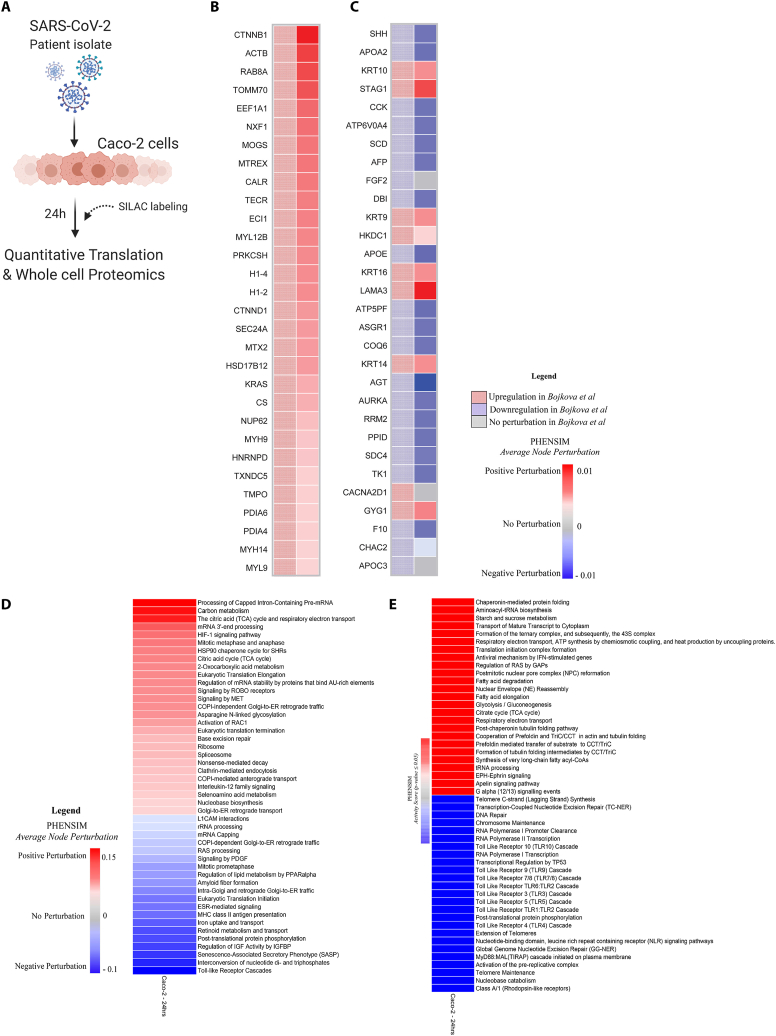
**PHENSIM proteomic pathway analysis in SARS-CoV-2-infected human host cells.** PHENSIM pathway analysis of the Caco-2 cell experiment was simulated *in silico* to reproduce *in vitro* results presented by Bojkova et al. at the 24 h time-point post SARS-CoV-2 infection **A)** Schematic representation depicting the experimental design as described by Bojkova et al. *in vitro:* the human colon epithelial carcinoma cell line, Caco-2 cells, were infected and monitored for 24hrs post SARS-CoV-2 infection. Naturally occurring heavy isotype SILAC labelling was used to quantify translational changes, as this method does not affect cellular behavior allowing for unbiased pathway analysis. Quantitative translation and whole cell proteomics by LC-MS/MS was performed [9]. **B&C)** Heatmaps depicting a representative subset of the 30 top differentially expressed proteins (FDR<0.05) involved in viral infection after 24hr SARS-CoV-2 infection ***B)*** as predicted by PHENSIM *in silico* (right column, solid squares), compared to expression results as determined by *Bojkova* et al. (left column, checkered squares) and ***C)*** as described by *Bojkova* et al. (left column, checkered) with side-by-side PHENSIM expression prediction for that protein (right column, solid). **D)** The heatmap shows the perturbation, as computed by PHENSIM, for a selection signaling pathways described as significant by *Bojkova* et al. in their analysis. **E)** The heatmap depicts the Top 50 pathways (25 Up- and 25 Down-regulated) significantly affected at 24h post infection, hence with p-value ≤ 0.05, according to PHENSIM prediction. In this case the Activity Score was taken into account. Color gradient reflects PHENSIM activity; the value of the activity score attributed to each pathway from blue (downregulation) to red (maximum upregulation). Caco-2; the human colon epithelial carcinoma cell line, SILAC; Stable Isotype Labeling by Amino Acids in Cell culture, LC-MS/MS; Liquid chromatography mass spectrometry, DEPs; Differentially expressed proteins, Max; maximum.

To validate PHENSIM on a proteomic level, we used our *in silico* approach to replicate the *in vitro* SARS-CoV-2 infection of human Caco-2 colon cells [9]. As viral genome copy number in cell culture supernatant and all viral protein levels assessed reached peak levels at 24h post infection, and the proteome underwent most extensive modulation [9], we focused on this 24h time-point for more in-depth comparison of protein expression and functional pathway analysis (Fig. 3). The PHENSIM simulation results obtained by leveraging the proteomic data 24hrs post SARS-CoV-2 infection are shown in Fig. 3. We provide an unbiased assessment by comparing the PHENSIM obtained Average Node perturbation *in silico*, to the 30 most perturbed proteins according to Bojkova et al. In order to compare *in vitro* to *in silico* protein expression levels a representative selection of relevant proteins involved in infection is depicted in the heatmaps in Fig. 3B and C. In Fig. 3B, proteomic perturbation of the top differentially expressed proteins (DEPs; n = 30) as predicted by PHENSIM (right, solid) is compared side-by-side to perturbation results from Bojkova et al. (left, checkered). Next, in Fig. 3C the top DEPs described by Bojkova et al. (right) is compared to PHENSIM predicted perturbation. Based on this selection of proteins we can denote a relatively high prediction rate for PHENSIM, although not all proteins are predicted to full accuracy. When quantifying the predictive power of PHENSIM on this protein-wide analysis, PHENSIM simulated results showed a predictive accuracy of 97.9% to the described *in vitro* proteomic data at 24hrs, where significant perturbation prediction was at 97.87% sensitivity and 97.96% specificity for this particular dataset (see Table 2).

**Table 2:** PHENSIM proteomic predicted values from *Bojkova* et al. 2020.

Time (hours)	All Proteins	Proteins in Meta-pathway	Predicted Percentage	Accuracy	PPV	Sensitivity	Specificity
***2***	5809	1914	6.95%	93.98%	95.45%	92.65%	95.38%
***6***	5809	1914	11.70%	93.75%	94.35%	97.66%	81.13%
***10***	5809	1914	10.45%	94.50%	95.27%	98.17%	77.78%
***24***	5809	1914	34.95%	97.91%	98.39%	97.87%	97.96%

*All Proteins*; number (N) of proteins quantified for each timepoint.

*Proteins in Meta-pathway*; number (N) of proteins present in KEGG, and therefore in the meta-pathway. Pre*dicted Percentage*; Percent (%) proteins for which PHENSIM could produce a prediction.

*Accuracy, PPV, Sensitivity, and Specificity;* the metrics used to compare our model with the actual proteomics data. PPV; Positive predictive value. *Bojkova* et al., Nature 2020 [9].

#### 2.3.2 PHENSIM proteomics: from *in vitro* to *in silico*

Next, to compare the Reactome-based *in vitro* functional pathway analysis [31] to our PHENSIM *in silico* approach, a representative selection of significantly affected pathways –correctly predicted by PHENSIM – is depicted in Fig. 3D. Pathways were selected according to the cellular mechanisms highlighted by *Bojkova* et al. [9]. The heatmaps in Fig. 3D&E shows the degree of perturbation (Fig. 3D) and the activity score (Fig. 3E; top to bottom), as predicted by PHENSIM, for each pathway. An in-depth analysis of proteomic pathways at 24hrs revealed distinct upregulation of various pathways involving cellular metabolism such as carbon metabolism, HIF-1 signaling and the Citric acid (TCA) cycle, as well as inflammatory and immune signaling pathways (Fig. 3D), previously described by *Bojkova* et al. as significantly perturbed. Additionally, to conduct an unbiased investigation at the pathway level, we next examined the pathways for which PHENSIM predicts a significant perturbation (i.e., with p-value ≤ 0.05). In Fig. 3E a selection of the Top 50 (25 up- and 25 down-regulated) pathways and their corresponding Activity Scores is depicted. Top upregulated pathways included antiviral mechanism by IFN-stimulated genes, and various metabolism pathways, respiratory electron transport pathway, fatty acid degradation, glycolysis/glyconeogenesis and the TCA cycle. Among the top downregulated pathways we observed an array of Toll-like Receptor signaling cascades, including the endosomal TLR7/8 and 9.

#### 2.3.3 PHENSIM predicts a metabolic signature in SARS-CoV-2 infection *in silico*

As a metabolic signature was identified by PHENSIM's proteomic *in silico* simulation of SARS-CoV-2 infection (Fig. 3D&E), we next assessed the degree of overlap between the perturbed genes of these metabolic pathways in order to reject the hypothesis that a common set of altered proteins is driving the significant perturbation of these closely related metabolic pathways. All metabolic pathways considered essential for SARS-CoV-2 infection according to the acquired Bojkova et al. data (Fig. 3) were included in the analysis (FDR-adjusted p-value <0.05) and a PHENSIM activity score was determined (see Supplementary Table S2). The affected general metabolic pathways showed very low degree of shared sub-pathway overlap. The Venn diagrams in Supplementary Fig. S4 show all possible intersections for the following top metabolic pathways: (i) Fatty acid degradation, Amino sugar and nucleotide sugar metabolism, Glycolysis/Gluconeogenesis, Citrate cycle (TCA cycle), and Purine metabolism; (ii) Glycolysis/Gluconeogenesis, Citrate cycle (TCA cycle), Purine metabolism, Carbon metabolism, and Pyrimidine metabolism.

### 2.4 PHENSIM drug repurposing strategy for COVID-19

**Fig. 4:**
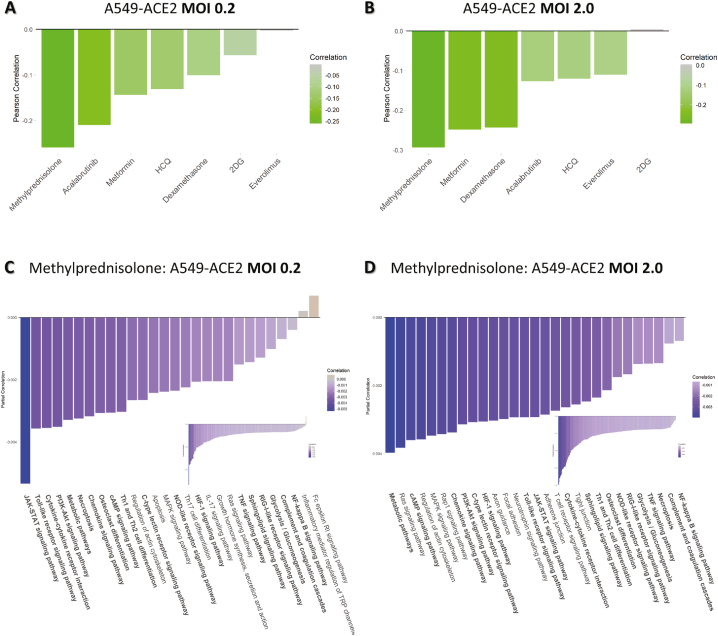
**Drug repositioning candidates for COVID-19.** We leverage our PHENSIM drug strategy approach to test candidate drugs for potential repurposing for COVID-19 treatment. Once a cell-specific viral signature is defined, it can be exploited to search for possible repositioning candidates by building a drug signature database. A Pearson correlation *p* (x,y) between the viral and drug signatures gives rise to a correlation score. Drug candidates having a positive effect on ameliorating SARS-CoV-2 infection have a negative correlation score (green) between viral and drug signature, whereas candidate drugs worsening disease correlate positively (red). Here we show distinct candidate drugs having a variable effect depending on the multiplicity of infection (MOI) of virus infection in A459-ACE2 expressing cells in **A)** low MOI 0.2 and **B)** high MOI 2.0. This analysis shows the modeling viral load dynamics and discerning what candidate could work best in low vs higher viral load. Resulted top pathways significantly affected by Methylprednisolone treatment are depicted for **C)** low MOI 0.2 and **D)** high MOI 2.0. Drug candidates represented here: Methylprednisolone, Metformin (mTOR-inhibitor), (Hydroxy)chloroquine (HCQ-CQ), Acalabrutinib (BTK-inhibitor), Dexamethasone**,** 2-Deoxy-Glucose (2DG) and Everolimus (mTOR-inhibitor). ACE2; angiotensin-converting enzyme, MOI; multiplicity of infection.

The next step in our PHENSIM approach is the employment of our drug strategy in order to test candidate drugs for potential COVID-19 repurposing. This approach takes advantage of existing knowledge on drug-related pharmacology and toxicology for rapid therapeutic selection [12]. As schematically described in Fig. 1, once a cell-specific viral signature is defined, it can be exploited to search for possible repositioning candidates by leveraging our select drug signature database. We used a Pearson correlation *p* (x,y) to compare the viral and drug signatures, which gives rise to a correlation score specific to that candidate drug, computed for SARS-CoV-2 infection in a particular setting. Here we set out to test a selection of hypothesis- and data-driven candidate drugs as shown in Fig. 4. One such drug, which regrettably failed to live up to its anticipated potential to effectively treat COVID-19 is the antimalarial drug hydroxychloroquine (HCQ), currently approved for rheumatologic implications although associated with cardiac toxicity [[32], [33], [34]].

Though the efficacy of corticosteroids in viral acute respiratory distress syndrome (ARDS) remains controversial, recent evidence on drugs such as Dexamethasone and Methylprednisolone are showing promise in COVID-19 [35,36]. Furthermore, the potential beneficial effects of blocking the mTOR pathway with use of mTOR-inhibitors such as Metformin, Everolimus or Rapamycin (the latter not evaluated here) in COVID-19 patients hasbeen hypothesized, however its effects on gene expression and distinct signaling pathways remain to be satisfyingly established. Considering targeting cell immunometabolism, 2-Deoxy-Glucose (2DG) was recently proposed as possible therapeutic in COVID-19 [9]. Lastly, therapeutic targeting of excessive host inflammation by inhibiting Bruton tyrosine kinase (BTK) in severe COVID-19 – for example the BTK-inhibtor Acalabrutinib –was recently described [37].

We evaluated a select set of candidate drugs for potential repurposing in SARS-CoV-2 infection as shown in Fig. 4 (and Supplementary Fig. S5). Here we show the results for A549-ACE2 cells at both a low (0.2, Fig. 4A) and high MOI (2.0, Fig. 4B). The other cell type scenarios are shown in Supplementary Fig. S5. The drug candidates having an ameliorating effect on SARS-CoV-2 infection *in silico* have a negative correlation score (green) between viral and drug signature, whereas candidate drugs worsening the disease phenotype have a positive correlation (red). Indeed, for both low and high viral load (MOI), Methylprednisolone, Metformin, Dexamethasone and Acalabrutinib positively correlated with the viral signature (green) which points to an effective therapeutic to target SARS-CoV-2 infection in A549 cells in the presence of ACE2, however, the order of the candidate drugs differed somewhat between the two viral loads. Using CoVariation analysis, we next looked at individual pathway contribution for each of the repositioning candidates evaluated here. The acquired Pearson correlation when comparing viral and drug-based signatures was dissected into components to show individual pathway contribution (see Fig. 4C and D and Supplementary Fig. S6). The overall effect of a candidate drug can be seen as the sum of the individually affected pathways, where anti-correlation is depicted in purple and positive correlation in orange. Here we use Methylprednisolone as an example for A549 cells expressing ACE2 receptor at low (0.2, Fig. 4C) and high MOI (2.0, Fig. 4D). Only significantly affected pathways are shown here. In Supplementary Fig. S6 we show pathway accumulation plots for the top 4 candidate drugs for A549-ACE2 at low and high MOI to illustrate the variation and effectiveness of the tested drug candidates and illustrate how the drugs were ordered based on best candidate (top; most pathways are anti-correlated (purple)), to least likely candidate of interest (bottom; mostly positively correlated pathways in orange). Some top anti-correlated pathways for Methylprednisolone, highly contributing to the final result of this drug candidate based on our PHENSIM analysis include the JAK-STAT pathway, the Toll-like receptor pathway (TLR), MAPK and PI3K-AKT signaling pathways. Next to similar pathways of importance affected for A549-ACE2 at both low and high viral MOI such as JAK-STAT, TLR, NOD-like receptor, RIG-I-like receptor and MAP-kinase (MAPK) signaling, Focal adhesion and Neurotrophin signaling pathway were among the top pathways observed at high viral load (MOI 2.0; Fig. 4D).

#### 2.4.1 PHENSIM methylprednisolone treatment of SARS-CoV-2 infected host cells *in silico*

**Fig. 5:**
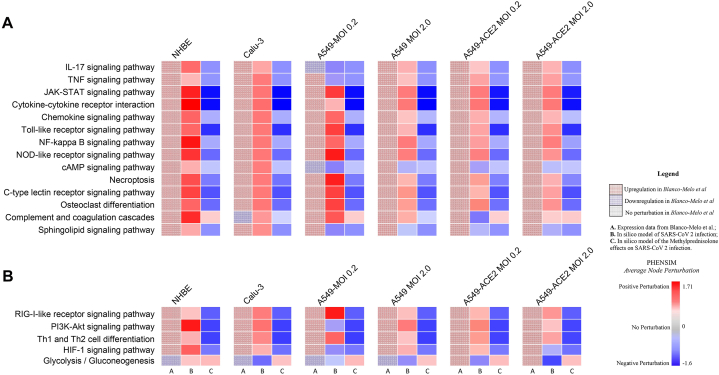
**Methylprednisolone inhibits key inflammatory and viral signaling pathways in host lung and airway cells after SARS-CoV-2 infection.** Heatmap depicts the effects of Methylprednisolone *in silico* in SARS-CoV-2 infection on select signaling pathways of interest as recently identified to be of importance by **A)** Catanzaro et al. 2020 and **B)** Draghici et al. 2020. From left to right for each cell-line depicted, ***column A***: pathway analysis results of SARS-CoV-2 infection *in vitro* as performed using the MITHrIL algorithm; ***column B:*** PHENSIM results of SARS-CoV-2 infection *in silico*; ***column C***: PHENSIM simulation results of Methylprednisolone on SARS-CoV-2 infected cells *in silico*. Color gradient depicts the average pathway perturbation as predicted in our PHENSIM *in silico* experiments for *column B&C*. NHBE; Normal Human Bronchial Epithelial cells, Calu-3; Cultured human airway epithelial cells, A549; Transformed lung alveolar cells, ACE2; angiotensin-converting enzyme, MOI; multiplicity of infection.

As a next step in our drug repurposing efforts, we simulate the simultaneous host-cell infection of SARS-CoV-2 and *in silico* treatment with Methylprednisolone (MP), hereby combining the drug action and pathogen infection on a host-cell, in order to further assess MP as a top candidate. We simulate SARS-CoV-2 viral infection and simultaneous MP treatment *in silico*, in order to more closely resemble the *in vivo* situation (see Fig. 5). In Fig. 5 we highlight PHENSIM's predicted perturbation of a select set of affected pathways during infection, as recently identified to be of importance by Catanzaro et al. 2020 (Fig. 5A) and Draghici et al. 2020 (Fig. 5B), and show the effects of MP treatment on these top affected pathways during SARS-CoV-2 infection in particular host-cells. The heatmaps in Fig. 5A&B depict the results of transcriptomic pathways analyses of host-cell SARS-CoV-2 infection, based on Blanco Melo et al. *in vitro* results (shown in left column A), and PHENSIM simulation *in silico* results (depicted in the middle column B), in comparison to MP treatment of *in silico* SARS-CoV-2 infected host cells (Fig. 5.; right column C). All identified upregulated pathways during infection were significantly inhibited by MP treatment, showing its known anti-inflammatory and immunosuppressive effects.

### 2.5 PHENSIM based drug repurposing validation

To evaluate the potential of our novel methodology for drug repositioning, we compared PHENSIM predictions to the *in vitro* drug screening performed by Stukalov et al. on A549-ACE2 cell lines [38]. As model of infection, we used our viral signatures obtained by the PHENSIM transcriptomic approach. The drug signatures were obtained using the L1000 dataset [39] on A549 cells. We subsequently performed simulation for 81 drug-concentration experiments using our PHENSIM transcriptomic drug repurposing approach. Therefore, we selected all repurposable drugs for our method (drugs showing an anti-correlated pattern with the disease) and compared the PHENSIM-obtained *in silico* results to the Stukalov et al. observed *in vitro* results *(see* Table 3). Furthermore, in our comparison we used L1000 concentration levels that best matched the concentrations used by Stukalov et al. [38]. In Table 3 we show that our results were significantly consistent with Stukalov et al.-reported *in vitro* results, observing approximately 67% (p = 0.009) of the repurposable drug candidates in common with the Stukalov et al. experiments.

**Table 3:** Drug candidate comparison using the PHENSIM repurposing approach.

	Number of repurposable drug/dosage combinations	Percentage of common drugs	p-value
Stukalov et al [38]	54		
PHENSIM repurposing A549 MOI 2.0	27	66.67%^a^	0.009
A549 MOI 0.2	35	62.86%^a^	0.001

a PHENSIM Comparison of potential repurposable drugs as described by Stuckalov et al. Nature 2021 [38].

## 3 Discussion

The current pandemic has accelerated the need for efficient and effective identification of potential drug candidates for COVD-19 pathogenesis. Knowledge on host-immune response to SARS-CoV-2 infection, however, remains limited with very few drugs approved to date. Various viable strategies and tools are rapidly arising to address this, where repurposing of existing drugs can offer a feasible mechanism of deployment (4). Here we introduce one such strategic approach, the *PHEN*otype *SIM*ulator, which allows the modeling of SARS-CoV-2 infection *in silico* and implementation to select promising candidates for further *in vitro* and *in vivo* analysis. We show that PHENSIM can effectively be used to *I)* predict the viral effects on cellular host-immune response and cellular pathways and *II)* evaluate a myriad of therapeutic strategies *in silico*.

As previously described, PHENSIM uses a probabilistic randomized algorithm to compute the effect of a particular biological scenario on gene regulation, protein expression, miRNA and metabolite involvement with use of KEGG meta-pathway analysis [20], and the addition of the Reactome [31]. Here we simulate SARS-CoV-2 viral infection based on publicly available data, to acquire a specific cellular SARS-CoV-2 signature. To verify a transcriptomic-based PHENSIM strategy, we compared the *in silico s*imulation results to publicly available transcriptomic data of SARS-CoV-2 infected cell lines [14]. We find that PHENSIM performs at high overall accuracy with a high PPV, sensitivity and specificity for all airway and lung-related cell lines evaluated. Key SARS-CoV-2 infection-related signaling pathways could be discerned as such, comprising the viral signature. PHENSIM predictive performance was further validated using our previously described MITHRIL transcriptomic pathway analysis [25], showing similar results. Interestingly, key signaling pathways in SARS-CoV-2 infection [8] were shown to be significantly perturbed in all cell lines studied *in silico* using PHENSIM, thereby offering promising potential molecular drug targets in COVID-19.

The PHENSIM strategy was also suitable for a proteomics/translatome-data based approach. PHENSIM simulation was compared to published SARS-CoV-2 infection-specific proteomic effects in host cell lines [9]. Comparing *in silico* results to proteomic data 24 h post infection showed high accuracy with a PPV, sensitivity and specificity well above 97%. Inhibition of several of these identified protein-associated pathways was previously shown to prevent viral replication in human cells [9].

We next used the transcriptomic-based PHENSIM approach to compare the viral signatures, computed with respect to model cell lines, to *in-silico*-derived drug signatures for a selection of drugs analyzed. Our overall correlation results show several potential drug repurposing candidates negatively correlating with SARS-CoV-2, varying from corticosteroids such as MP (already approved for treatment of COVID-19 patients), biologicals such as BTK-inhibitors that are currently being studied in clinical trials [37] to metformin [40]. Individual signaling pathway contribution to the observed correlation score could be further delineated for each individual drug, providing potential specific targets for in depth analysis and potential for pathway-specific therapeutic targeting. As expected, the individual pathways most targeted by the *in silico* drug interventions (Fig. 4C&D) were similar to pathways found most perturbed by PHENSIM during host transcriptomic response to SARS-CoV-2 viral infection (Fig. 2C&D), emphasizing their potential therapeutic effects. HCQ, although hypothesized early on to be a good potential candidate to treat COVID-19, has not proven effective *in vivo* [41]. The exact reason why HCQ has failed in COVID-19 remains to be fully understood. Interestingly, COVID-19 is associated with a variety of hematologic complications [42], and increased HCQ use during the COVID-19 pandemic has induced the emergence of methemoglobinemia, including tissue hypoxia and reduced oxygenation [43,44] amongst others. Evidently, evaluating the risk-benefit ratios – drug safety and efficacy – is crucial when selecting drugs to be repurposed for COVID-19 [12], which particularly holds true for HCQ [33,34,45].

In Fig. 4, we depict our drug repurposing PHENSIM approach that functions as a screening tool for initial *in vitro* drug candidate screening, based on the anti-correlation of the viral and drug signatures, and gives a particular score for each candidate; a negative correlation constitutes higher *in vitro* potential for that particular drug. This broader correlation approach described in Fig. 4 and Supplementary Figs. S5 and S6 can be used to screen large sets of candidate drugs. Next, as depicted in Fig. 5, a more dynamic and extensive analysis can be simulated, in order to simulate the interaction between the SARS-CoV-2 host-cell infection (column B) and subsequent *in silico* treatment with a candidate drug, here Methylprednisolone (column C). Although Methylprednisolone is a known broad-spectrum corticosteroid, with clear anti-inflammatory and immunosuppressive effects (as shown in Fig. 5), complete inhibition of these crucial immune signaling pathways might not be beneficial to COVID-19 patients at every stage of disease; which has been described in clinical practice. Other, more targeted drug candidates might be more beneficial to the overall functioning of the patient's immune system during the fight and recovery from COVID-19. Indeed, our detailed approach can be implemented for all other top candidates, for further in-depth evaluation of their *in vitro* potential. However, we should bear in mind that the simulation is simultaneous (both virus and drug) and not completely reflective of a sequential treatment of a drug during infection. We are currently leveraging our simultaneous approach to evaluate the use of Metformin in COVID-19 in more detail.

Drug repurposing towards COVID-19 is challenging, but also poses many new opportunities. Several innovative approaches have been used varying from structure assisted computer designed mini inhibitors of receptor binding domain (RBD) binding [46], inhibitors of viral key enzymes like Mpro [10,47,48], machine learning models predicting compound protein inhibiting activity [49] to infected cell-based assays drug screening [50,51]. Using computational tools, such as PHENSIM, allows for safe exploration of potential candidate drugs and uses previously acquired knowledge from biomedical databases to narrow the scope of possible viable biomarkers and druggable targets. One of the clear advantages of PHENSIM is a more effective selection of hypothesis driven drugs, before initiating extensive, time-consuming and costly *in vitro* experiments that should eventually provide the basis for clinical studies. PHENSIM requires on average (depending on data availability) about 3 h of simulation time. Another interesting possibility enabled by our approach is the potential capability to not only simulate the effect of a single drug, but also drug combinations. This expansion of PHENSIM is currently being developed (see Methods section; *data availability).* By making use of not just viral targets but also host proteins and structured pathways in the computation of the PHENSIM viral signature, we broaden the scope of potential drug targets with the added advantage that these are less prone to resistance development [52]. Here we simulated a select set of candidate drugs for repurposing in COVID-19, however, many candidates can be further evaluated by our PHENSIM system *in silico* in the near future. We can also identify additional candidates based on the SARS-CoV-2 viral signature acquired by PHENSIM and recent data on IFN-involvement in COVID-19 [reviewed in Ref. [53]], targeting the JAK/STAT pathway using Baracitinib – approved for moderate to severe arthritis [54] – recently shown to reduce time-to-recovery for hospitalized COVID-19 patients in combination with Remdesivir [55], however, caution is warranted [56].

One of the advantages of the PHENSIM algorithm is the capacity to add on information of particular genes of interest (as specific knowledge becomes available) to the original simulator, if absent in KEGG. In the case of SARS-CoV-2 infection, the absence of some important genes involved in infection of host cells in the KEGG database, was considered a limitation. One gene in particular is Basigin (BSG) (also known as the CD147) gene. The importance of CD147 in SARS-CoV-2 infection in spike-protein (SP) binding and viral infiltration of host cells has been described [57]. In our current approach we model SARS-CoV-2 infection *in silico* with the addition of CD147 gene into KEGG, hereby developing an *in silico* ‘knock-in’ of the CD147 gene, in order to investigate the role of this extracellular matrix metalloproteinase inducer (EMMPRIN) in COVID-19 (see Supplemental Table S1).

As discussed, the *in silico* model presented here provides an interesting framework that can be constinously developed and expanded further, achieving a more complete cell signature with input of (newly) available data on processes such as cell-cell communication through ligand-receptor complexes [58] or viral immune evasion e.g. Ref. [59]. As most *in vitro* studies are performed on cell lines, tissue tropism characteristics of viral infection seem key to better understanding viral activity [60]. The same model could be adapted to study specific cells involved in viral infection like tissue-specific epithelial cells and immune cells (e.g. T cells and NK cells) [61,62]. Moreover, many interesting avenues can potentially be explored using PHENSIM, such as modeling immune-related effects of this pathogen and others, in distinct tissue-specific non-immune epithelial cells, stem cells, and beyond [15,[61], [62], [63]]. The system can be further adapted to include new data gathered on the viral translational landscape related to newly discovered open reading frames (ORFs) and potential novel polypeptides/proteins and infectivity potentiating cell surface structures like neuropilin [64,65]. Interestingly, integration of all the aforementioned schemes could potentially yield novel and effective drug targets [66].

Here we show distinct candidate drugs having a variable effect depending on the multiplicity of infection (MOI) of virus infection in A549-ACE2 expressing cells in low (MOI 0.2; Fig. 4A) and high MOI (2.0; Fig. 4B). As shown, the sequence of top candidate drugs for repurposing is slightly different depending on the cell-type, viral load (MOI), and expression of the viral entry receptor ACE2 (see Fig. 4 and Supplementary Fig. S2). This speaks to the variability of how the virus might affect specific cell types and tissues, even within the same organ system such as the bronchial (NHBE), airway epithelial (Calu-3) and lung alveolar (A549) cells e.g., pointing to the difficulty in specifically targeting this viral infection therapeutically. It will also depend on the stage of infection and disease state, as to which course of treatment or combination of treatments will be optimal.

To further validate the power of PHENSIM, we leveraged a recently published *in vitro* SARS-CoV-2 drug screening [38]. Our results were significantly consistent with Stukalov et al-reported *in vitro* results (Table 3). Indeed, we observed that approximately 67% (p = 0.009) of our selected *in vitro* repurposable candidates were also reported as viable candidates in the Stukalov et al. experiments. Observed differences, however, may be attributable to the inevitable differences between the compared data. It should be considered that Stukalov et al. perform their *in vitro* experiments on A549-ACE2 cells [38], while L1000 data concerns A549 cells [39]. Additionally, *in vitro* viral inhibition assays of Stukalov et al. were performed at MOI 3.0 [38] whereas our *in-silico* models rely on infection data at both MOI 0.2 and 2.0. Interestingly, our repositioning approach showed that the B-RAF inhibitors Sorafenib, Regorafenib and Dabrafenib and the JAK1/2 inhibitor Baricitinib, which are commonly used to treat cancer and autoimmune diseases [67,68], led to a significant increase in virus infection. Our results reveal a slight anticorrelation of Tirapazamine, an inducer of DNA damage at a concentration of 2.22 μM on A549-ACE-2 MOI 2.0 cells, reflecting the findings of Stukalov et al. Furthermore, we observe potential effects for mTOR inhibitors such as Ramapicin, to which we here compared Sirolimus.

As demonstrated by our results, we believe that the PHENSIM system provides a simulation for a multitude of powerful systems biology functions and implements them easily and efficiently. PHENSIM is a simulation algorithm which follows the biological processes modeled by pathways. Therefore, PHENSIM is able to make a prediction of such processes and not only of the final effect, going beyond methods based on pathway enrichment. Furthermore, since pharmacological treatments may depend on the state of biological processes, PHENSIM may be of more appropriate use in this context. Comparison with other simulation algorithms such as BIONSI [17,18] has shown excellent performance by PHENSIM [20]. PHENSIM creates and builds on interpretable and intervenable mechanistic bio-chemical models, rather than combinatorial and statistical “black-box” models for joint stationary distribution of biological data, as in, say protein-protein interaction (PPI) networks, Graphical or Deep-net models.

PHENSIM gives rise to feasible validation and comparison of *in vitro* and *in vivo* experimental data [8,9], gives insight into drug efficacy [9,36,52], tracks specific host signal transduction pathways [8], *in silico* testing of single drugs and drug combinations and further delineation of future targets (e.g. CD147) and identification of specific pathways of action of both pathogen and therapeutic compound in healthy and infected systems. For cost efficiency, validated predictive methods and assays for early elimination of potential drug candidates are of great value [69]. The overall efficiency (time, costs, safety) prompts to suggest implementing PHENSIM not only in viral acute pandemic settings [70], but in additional curative and non-curative diseases, especially complex chronic disorders, where both *in vitro* experiments and clinical trials are time-consuming or impossible to reduce to practice. Optimally leveraging the power of pathway analysis by simulating host cell and tissue-specific infection and performing *in silico* drug selection, has a tremendous potential beyond COVID-19, with applicability to high global burden communicable diseases, translatable to pathogens of viral, bacterial and fungal origin, and potentially chronic disease such as inflamm-aging and diabetes. In conclusion, our PHENSIM approach will enable more effective *in vitro* experiments resulting in more rapidly initiated clinical trials and accelerated regulatory review of already pre-selected drugs with a high repurposable potential.

## 4 Limitations of study

The potential absence of some unknown important genes involved in SARS-CoV-2 infection of host cells in the KEGG databaseis a limitation of this study. Additionally, PHENSIM-determined potential candidates were based on *in vitro* and cell-specific data, and will still need to be evaluated further in an *in vitro* and *in vivo* setting, as exemplified by HCQ which has not proven to be effective *in vivo*. Furthermore, our analysis is restricted to simulation *of in vitro* cell line data for specific cell types.

## 4 Star methods

Detailed methods are provided in the online version of this paper and include:
- KEY RESOURCES TABLE
- RESOURCE AVAILABILITY
o Data and Code Availability:

All input data, raw images, and source codes for PHENSIM are available athttps://github.com/alaimos/phensim-covid19. Website: https://phensim.atlas.dmi.unict.it/[20].

o Lead contact: **Bud Mishra:** mishra@nyu.edu. Courant Institute of Mathematical Sciences Room 405, 251 Mercer Street, New York, NY 10012.

## Author contributions

Conceptualization, design, drafting and revision of the manuscript: NIM, RVR, SA, BM, AJD, AF.

Concept, design, and revisions of the manuscript: EB, AS, JACB, AP.

Methodology: SA, AP; Writing original draft: NIM; Supervision: NIM, BM, AJD, AF.

## Competing interests

All authors declare no competing interests.

## Data availability

All input data, raw images, and source codes for PHENSIM are available at https://github.com/alaimos/phensim-covid19. Website: https://phensim.atlas.dmi.unict.it/ [20].

## Funding

N.I.M. was funded in part by a fellowship award from the Netherlands-Caribbean Foundation for Clinical Higher Education (NASKHO). S.A., A.F. and A.P. have been partially supported by the MIUR PON research project BILIGeCT “Liquid Biopsies for Cancer Clinical Management”.

## TRANSPARENT METHODS

### Data Retrieval and Processing

To determine the efficacy of our methodology, we used several datasets obtained in the context of SARS-CoV-2 infection. We exploited the transcriptomics data published in Blanco-Melo et al. [14] [GSE147507] Campo 14 and proteomics data from Bojkova et al. 2020 [9] for the construction of the viral models (transcriptomic and proteomic viral models, respectively). Furthermore, we gathered drug-target interactions data from Drugbank [72], and drug expression data from the LINCS L1000 dataset for the A549 cell line [39]. More in detail, the Blanco-Melo et al. dataset comprises RNA-seq data of infected vs. mock-treated cell lines from human and ferret. The data were collected using the Illumina NextSeq 500 sequencing platform [14]. In our analysis, we focused solely on human cell data. Four cell lines were evaluated: primary human lung epithelium (NHBE), transformed lung alveolar (A549) cells, transformed lung alveolar cells (A549) transduced with a vector expressing human ACE2, and transformed lung-derived Calu-3 cells. For all cell lines, sequencing data of biological triplicates were obtained from mock-treated or SARS-CoV-2 infected experiments. Furthermore, for both A549 cell lines, different MOIs (multiplicity of infection) were used at low (0.2) and high MOI (2.0). Following the same procedure outlined in Blanco-Melo et al., raw counts were normalized and analyzed for differential expression using the DESeq2 pipeline. All genes with an FDR-adjusted p-value<0.05 (Benjamini-Hochberg FDR [73]) and absolute LogFC>0.6 were considered differentially expressed. Non-expressed genes for a specific cell line were defined as genes that showed an average read count lower than 10. The Bojkova et al. 2020 dataset comprises proteome measurement by LC-MS/MS of control vs. SARS-CoV-2-infected human Caco-2 cell lines [9]. All cell lines were analyzed in triplicates (n = 3) at 2, 4, 10, and 24h. Log2-ratios between infected and normal differentially expressed proteins (DEPs) (p-value<0.05) were used as input for the simulation algorithm. Non-expressed proteins for the Caco-2 cells were taken from the Human Protein Atlas (using the query “celline_category_rna:CACO-2; Not detected”). Since PHENSIM uses Entrez Gene Identifiers, we mapped all proteins to their gene, yielding 5809 mapped proteins. Of these 5809 mapped proteins, we could find only 1914 in our meta-pathway.

Finally, from the LINCS L1000 dataset for the A549 cell line, we downloaded Level 5 transcriptomics signatures of all drugs tested in Stukalov et al. (27 drugs) [38]. For each drug, we gathered only samples with concentration values closely matching Stukalov et *al*., yielding 81 drug-concentration transcriptomic experiments. For each sample, we determined differentially expressed genes from Level 5 data z-scores using the thresholds detailed in the LINCS L1000 pipeline (z<−1.96 for down-regulated DEGs and z>1.96 for up-regulated DEGs).

### Outline of the methodology

Our approach defines a protocol for the in-silico simulation of emerging pathogen infection aimed at generating a hypothesis for drug repositioning. The protocol is based on the PHENSIM algorithm. PHENSIM is a systems biology approach for simulating the effect of biomolecule alteration in a specific cellular context using the KEGG and REACTOME [31] meta-pathway, a model of the physiological cell, accounting for pathway crosstalk. More details on the PHENSIM algorithms are described in the “The PHENSIM approach” section.

Our protocol starts by finding a representation of the pathogen within our meta-pathway, allowing us to perform simulations. Since, for a novel pathogen, interactions with the host genes are mostly unknown, we approximate this with transcriptomics or proteomics data. These data are used to perform a simulation on the meta-pathway to obtain a signature of the pathogen effect on the host phenotype (see section “Deriving signatures from PHENSIM” for more details).

Next, we employ the same approach to drug expression data to derive a database of candidate treatment signatures by performing simulations for each drug on the same cellular context of the pathogen. Therefore, we match the pathogen signature against each drug signature using a Pearson correlation function, yielding a score for each drug. The viable repurposing hypotheses are drugs showing a negative correlation score since it represents drugs with an opposite effect to the virus on the host phenotype in our meta-pathway representation. We also compute a p-value for each prediction to assess their statistical significance, which is corrected for multiple hypotheses using the Benjamini-Hochberg method (see section “Generation of repurposing hypothesis” for more details).

Finally, for a user-defined selection of candidates, predictions are investigated by decomposing the Pearson correlation score to determine which pathways are the most affected by the drug and by performing a combined drug/pathogen simulation (see section “Investigating repurposing hypothesis” for more details).

### The PHENSIM approach

PHENSIM is a systems biology approach, simulating the effect of the alteration of one or more biomolecules (genes, proteins, microRNAs, or metabolites) in a specific cellular context using our KEGG (Kyoto Encyclopedia of Genes and Genomes) and REACTOME meta-pathway cell lines [31,38]. The meta-pathway concept, introduced by us previously [74], has been devised as a model of the physiological cell, accounting for pathway cross-talk in the analysis. All KEGG and REACTOME [31] pathways are merged in a single graph through common nodes. Therefore, the meta-pathway is a graph in which the nodes represent molecular entities (genes, metabolites), and the edges are the known biological interactions present in the KEGG and REACTOME [31] databases. Since we want to build a model of the physiological state of a cell, all pathways representing diseases and disorders are not considered. The meta-pathway is further completed by adding validated miRNA-targets downloaded from miRTarBase (release 8.0), miRecords, and TF-miRNA-interactions obtained from TransmiR (release 2.0) [75].

To start the simulation, PHENSIM requires a set of biomolecules as input, their direction of deregulation (activation/up-regulation or inhibition/down-regulation), and a set of inactive biomolecules in the cellular context (cell lines, tissue, e.g.). The algorithm uses these details to compute synthetic Log Fold Changes (LogFC). Synthetic LogFCs are calculated by sampling the normal distribution fitted to the actual LogFCs determined over a large set of transcriptomics experiments, as described previously [20]. Such values are then propagated within the meta-pathway, using the MITHrIL (Mirna enrIched paTHway Impact anaLysis) pathway perturbation analysis [25]. MITHrIL can predict how local change on a gene expression can affect the cellular environment by computing, for each gene of the meta-pathway, a “perturbation factor.” Such perturbation reflects its expected change of expression/activity (negative/positive for down-/up-regulation, respectively). Therefore, given a node x of the meta-pathway at i-th simulation step, its perturbation $P_{i}\left(x\right)$ is computed, following equation 3 of Alaimo et al., 2021, as: $P_{i}\left(x\right)=\Delta E\left(x\right)+\sum_{u\in U\left(x\right)}\frac{w\left(u,x\right)}{\sum_{d\in D\left(x\right)}w\left(u,d\right)}P_{i}\left(u\right)$, where U(x) and D(x) are the set of upstream and downstream nodes of x in the meta-pathway, respectively, and w(j,k) is a weight reflecting the type of interaction between nodes j and k. In PHENSIM, we use w(j,k)=1 for all activating interactions, w(j,k)=−1 for all inhibiting ones.

Then, these results are collected and synthesized using two values: the “Average Perturbation” and the "Activity Score" (AS). Given a node, the average perturbation is the mean for its perturbation values computed at each simulation step. It reproduces the expected change of expression for the entire process. The function of AS is twofold: 1) the sign gives the type of predicted effect (activation (+); inhibition (−)), 2) the value is the log-likelihood that this effect will occur.

More in detail, let $P_{i}\left(x\right)$ be the perturbation of a given node x of the meta-pathway at the i-th step of the simulation, and T the total number of simulation steps. The average perturbation of x, $\bar{P}\left(x\right)$, as $\bar{P}\left(x\right)=\frac{1}{T}\sum_{i=1}^{T}P_{i}\left(x\right)\text{.}$ The activity score of x, AS(x), can be determined, following equation 5 of Alaimo et al., 2021,as:

$$AS\left(x\right)=\left\{ \begin{array}{cc} \log_{2}\left(\frac{\mathrm{Pr}\left(x=1\right)}{1-\mathrm{Pr}\left(x=1\right)}\right) & if\;\mathrm{Pr}\left(x=1\right)>\left(1-\mathrm{Pr}\left(x=1\right)\right)\\ -\log_{2}\left(\frac{\mathrm{Pr}\left(V_{i}=-1\right)}{1-\mathrm{Pr}\left(V_{i}=-1\right)}\right) & if\;\mathrm{Pr}\left(x=-1\right)>\left(1-\mathrm{Pr}\left(x=-1\right)\right)\\ 0 & if\;\mathrm{Pr}\left(x=0\right)>\left(1-\mathrm{Pr}\left(x=0\right)\right) \end{array}\right.$$

where Pr(x=v) is the empirical probability estimated as:

$$=\frac{\left|\left\{p\in P_{i}\left(x\right)|sign\left(p\right)=v\right\}\right|}{T}$$

Finally, together with AS, PHENSIM also calculates a p-value which determines how biologically relevant the predicted alteration is for the phenomena being simulated (See section “P-values computation and False Discovery Rate” of Alaimo et al., 2021).

All p-values computed by PHENSIM are corrected for multiple hypotheses using the q-value algorithm [76]. To determine this probability, PHENSIM randomly selects genes in the meta-pathway and simulates this random set. By repeating this procedure (N = 1000 for our simulations), it is possible to empirically estimate the probability that a node has a higher activity score than the observed one. For this reason, we can employ such a value to determine which alterations are most specific for a particular infection, gaining novel hypotheses on the molecular action of the pathogen.

### Deriving signatures from PHENSIM

As stated in the “Outline of the methodology” section, our approach defines a protocol for the in-silico simulation of emerging pathogen infection to generate a repurposing hypothesis. First, we find a pathogen/drug representation on the meta-pathway, which allows us to run simulations using PHENSIM. Therefore, we use these simulations to generate a hypothesis.

For a novel pathogen like SARS-CoV-2, interactions with the host genes might be unknown. Therefore, we approximate the viral model by employing expression data from pre-/post-infection samples. The rationale is that differentially expressed genes (DEGs) represent the downstream effects of the viral infection on the host.

Therefore, we compute DEGs between pre- and post-infection samples and extend the meta-pathway by adding a new node representing the virus. Then, the viral node is connected to each DEG with an activating (/inhibiting) edge if its LogFC computed between post- and pre-infection is positive (/negative). Finally, we can run a PHENSIM simulation on the meta-pathway by giving the upregulation of the viral node as input (Fig. 1a and b).

To build the pathogen signature, which represents the simulated effect of the pathogen on the host phenotype, we use pathway endpoints. An endpoint is a biological element in a pathway whose alteration, based on current knowledge, affects the phenotype in a specific way [25]. In KEGG, these genes are reported for each pathway (see Fig. 1c for an example).

Therefore, given the output of a simulation, we call its signature the collection of the activity score computed for each endpoint node. When building the simulation signature, we do not use any p-value. Indeed, we need to consider not only the alterations, which are the most specific for a specific simulation, but also the alterations caused by any cellular response. Since the p-value represents the biological relevance for the phenomena that is being simulated, we can ignore its value to build the signature.

The same approach can be used to derive signatures for drugs activity on the host phenotype. In this case we add a node to the meta-pathway representing the drug. The node is connected to the genes in the meta-pathway by employing its targets, identified through databases (i.e., Drugbank or Pubchem) and literature (Pubmed) searches, or by computing DEGs between treated vs untreaded cell-lines, when targets are unknown. Then, the drug node is connected to each DEG/target with an activating (/inhibiting) edge using the same approach described previously. Finally, we run a PHENSIM simulation on the meta-pathway by giving the upregulation of the drug node as input (Fig. 1d and e) and collect the signature of this simulation (Fig. 1f).

Furthermore, for each drug we compute random models to empirically estimate repurposing p-values. Let T be the set of targets for a drug. First, we select a random set of targets of the same size as T. Therefore, we perform a PHENSIM simulation with the random set using the same alterations of the drug. Finally, we collect the signature as described above. This procedure is repeated for each drug to gather N random signatures (N = 1000 in our experiments), that are stored for the p-value computation phase.

### Generation of repurposing hypothesis

Our drug repurposing methodology is based on a similarity search performed on the drug signature database. Given a pathogen profile computed with PHENSIM, we use Pearson correlation to scan through each drug profile. This procedure yields a ranking on each drug in the range [−1; 1], where negative values indicate that the virus alteration profile is opposite to the drug and positive values indicate the reverse; drugs with a negative correlation are considered viable hypothesis. In our experiments, we employ a Pearson correlation function to run the similarity search. Since PHENSIM is based on MITHrIL pathway perturbation analysis, which computes results in a log-linear space [25], we can assume a Pearson correlation is suitable to determine similarity between the viral and drug signature. A key characteristic of this approach is the capability to simulate both single and drug combinations. Furthermore, PHENSIM also provides a framework for extending pathways by adding new nodes and edges coming from results in the literature as well as other reputable sources.

More in detail, let D and V be drug and pathogen alteration profiles, respectively. That is, D [e] is the activity score of endpoint “e” computed by PHENSIM for a drug simulation, and V [e] is the activity score for the same endpoint in the pathogen simulation. Pearson correlation ρ(D,V) can be written as equation (1): $\rho \left(D,V\right)=\frac{\sum_{e}\left(D\left[e\right]-\bar{D}\right)\left(V\left[e\right]-\bar{V}\right)}{\sigma \left(D\right)\cdot \sigma \left(V\right)}$, where $\bar{D}$ and $\bar{V}$ are the means of D and V, respectively, and σ(⋅) is the standard deviation (Fig. 1g and h).

To determine the statistical significance of the results, we use an empirical approach based on a bootstrapping procedure known as permutation test. First for each drug D, we build N random signatures $D_{i}^{\prime }$ as described in section “Deriving signatures from PHENSIM”. Next, for each signature we compute the Pearson correlation coefficient against the pathogen profile, $\rho \left(D_{i}^{\prime },V\right)$. Finally, we count the number of times we obtain a greater correlation to empirically estimate the p-value as:

$$p=\frac{\left|\left\{D_{i}^{\prime }|\left|\rho \left(D_{i}^{\prime },V\right)\right|>\left|\rho \left(D,V\right)\right|\right\}\right|}{N}$$

Finally, p-values are corrected for multiple hypotheses using the Benjamini-Hochberg procedure.

### Investigating repurposing hypothesis

To further explore the biological details leading to the prediction of interesting candidates, we applied a twofold strategy: (i) we assess whether an interesting drug candidate targets relevant infection processes by decomposing the Pearson correlation in terms of pathways, and (ii) we perform a combined pathogen/drug PHENSIM simulation to predict how the infection processes identified in the previous step are altered.

Therefore, given a pathway P, we can decompose the total Pearson correlation by summing over all components belonging to its endpoints to estimate how much it contributes to the final correlation value. More in detail, the partial correlation $\hat{\rho }\left(D,V,P\right)$ can be computed as equation (2): $\hat{\rho }\left(D,V,P\right)=\frac{\sum_{e\in P}\left(D\left[e\right]-\bar{D}\right)\left(V\left[e\right]-\bar{V}\right)}{\sigma \left(D\right)\cdot \sigma \left(V\right)}$, where D and V are drug and pathogen alteration profiles, respectively, D [e] is the activity score of endpoint “e” computed by PHENSIM for a drug simulation, V [e] is the activity score for the same endpoint in the pathogen simulation, $\bar{D}$ and $\bar{V}$ are the means of D and V, respectively, and σ(⋅) is their standard deviation. A significant feature of this partial correlation approach is that we obtain the total correlation by summing up all values for each pathway P

Next, we gather all the results for each pathway and review them to identify interesting processes that to the current knowledge should be targeted by a pathogen infection.

Finally, to further evaluate whether the results of the correlation could be confirmed by PHENSIM, we devised a strategy to simultaneously simulate drug action and pathogen infection on a host cell line. First, we collected DEGs between pre- and post-infection samples as described in the previous section. Then, given a drug, we gather its known targets and their alterations (up/down-regulations caused by the drug) through databases (i.e., Drugbank or Pubchem) and literature (Pubmed) searches, or through treated vs untreated DEGs experiments (L1000). Therefore, we extend the meta-pathway by adding two nodes, representing the virus and the drug, respectively, that are connected to the other elements in the meta-pathway following the procedure detailed in the “Deriving signatures from PHENSIM” section. Finally, we can run a PHENSIM simulation by giving as input the simultaneous upregulation of both virus and drug nodes (results depicted in Fig. 5).

### In-silico evaluation of the pathogen model: application to the SARS-CoV-2 infection

To determine the efficacy of our model *in silico*, exploiting the recent SARS-CoV-2 pandemic, we used several datasets obtained in the context of the infection. We focused on two fronts: i) the ability of PHENSIM to predict genes altered in the expression data, and ii) the ability to predict the correct direction of the alteration. In detail, we define as altered all genes having an absolute LogFC>0.6. The type of the alteration is given by the sign of the LogFC (+LogFC for upregulation, -LogFC for downregulation). Predictive power of PHENSIM was assessed by means of Positive Predictive Value (PPV), Sensitivity, and Specificity. The PPV is the proportion of true positive results with respect to all positive predictions, the sensitivity is the percentage of true positives with respect to the entire population, and the specificity is the percentage of true negatives with respect to all negative cases. For our analysis we exploited two independent datasets, the RNA-seq data of Blanco-Melo et al. and the proteomics data of Bojkova et al. yielding two separate models, a transcriptomic and a proteomic model. We separately evaluated the results obtained from the two approaches.

Prior to running all simulations, we verified if viral entry points were present in our meta-pathway to better represent viral activity. SARS-CoV-2 is said to invade host cells via these two receptors: angiotensin-converting enzyme 2 (ACE2) and CD147 (also known as Basigin or EMMPRIN) [57]. In KEGG, the latter gene is missing and to extend our simulation model, a new node, representing CD147 was added and connected with its known interactions and downstream nodes according to literature (Supplementary Table S1). In Supplementary Table S1, we report a list of up- and downstream genes added to our simulations and their respective references containing interaction details. CD147 is a transmembrane protein of the immunoglobulin super family, expressed in many tissues and cells, acting as the main upstream stimulator of matrix metalloproteinases (MMPs) and playing a crucial role in intercellular recognition [77]. Over the last decade, several groups have shown that CD147 acts as a key molecule in the pathogenesis of several human diseases including infectious diseases (HIV, HBV, HCV, KSHV) [77], and it has now been posed to recognize and internalize/endocytose SARS-CoV-2 in certain cell types [57].

To find whether the predictions made by our simulator were biologically relevant, we compared the results with published *in vitro* experiments from Blanco-Melo et al., Bojkova et al. and Draghici et al. [9,14,36]. First, we compared the results from Blanco-Melo et al. with our in-silico predictions for NHBE, Calu-3, A549 (MOI 0.2 and 2) and A549 transduced with ACE-2 (MOI 0.2 and 2) cells. Transcriptomics data for all cell lines were collected from GEO dataset GSE147507 and Log2-LogFCs were computed. Next, LogFCs were compared with our predicted Activity Scores (AS) by accounting for their direction of perturbation. We compared our predictions with the genes that Blanco-Melo et al. reports as important in the antiviral host-response to SARS-CoV-2. Furthermore, using an unbiased approach and to verify the accuracy of PHENSIM, we assessed the top-10 upregulated and top-10 downregulated genes for each cell-line. Finally, we assessed our viral simulation with results from Draghici et al., 2020 and Catanzaro et al., 2020 [8,36].

Pathway analysis was applied to the transcriptomics data to determine which biological processes were altered by the viral infection. We used 4 pathway analyses approaches to assess the most impacted pathways: 1) MITHrIL, 2) SPIA, 3) Reactome Pathways and 4) Gene Ontology Enrichment analysis. MITHrIL pathway analysis was performed as described in Alaimo et al., 2016 [78]. We used the LogFC of DEGs for all cell lines to perform MITHrIL perturbation analysis on the KEGG-only meta-pathway. Therefore, all values were aggregated on a pathway basis to compute an Accumulator and a p-value. Finally, p-values were adjusted for multiple hypotheses using the Benjamini-Hochberg FDR correction. Results were filtered by an FDR-adjusted p-value of 0.05 and ranked using the Accumulator. The top-25 significant pathways were reported in Fig. 2D&E. SPIA analysis was performed as previously described by Tarca et al., 2009 [79]; the LogFC of DEGs and ranked pathways were calculated using FDR-adjusted p-values as computed by SPIA. Pathways with a p < 0.05 were considered significant.

Finally, to further expand our understanding of the biological processes affected by the infection, we performed enrichment analysis on both Reactome Pathways, using the ReactomePA package [80], and Gene Ontology, using the GOfuncR package [81]. All results produced by the 4 pathway methodologies were collected and considered significant with an FDR-adjusted p-value<0.05 (Supplementary Fig. S3 and Supplementary material).

Specifically for proteomics data, we combined our PHENSIM analysis with an enrichment analysis to determine if a prediction made by our methodology was based on adequate data (Supplementary Table S2). More in detail, for each time point and each pathway, we compared the number of altered proteins predicted by PHENSIM to the expected number of altered proteins using a hypergeometric distribution. This analysis yielded an enrichment p-value combined with PHENSIM one using the Fisher's Method [82] due to their independence. P-values were corrected for multiple hypotheses using the Benjamini-Hochberg correction, and all pathways with a p-value<0.05 were considered significant for further analyses.

Since Bojkova et al. reported their results using Reactome Pathway analysis [9,31], to compare with MITHrIL and SPIA pathway analysis, we selected all pathways corresponding to the SARS-CoV-2 highlighted cellular processes (translation, splicing, carbon metabolism and nucleic acid metabolism, for instance) (Fig. 3D). Finally, since we found many similar metabolic pathways significantly affected *in silico* as described *in vitro* by Bojkova et al. we aimed to determine if a core set of proteins was common between pathways; results are displayed in the VENN diagrams in Supplementary Fig. S4 and Table S2.

### In-silico evaluation of the repurposing hypothesis

To assess the performance of our drug repurposing approach, we compared predictions acquired using our pipeline to build both pathogen and drug signatures (see Fig. 1), with the *in vitro* drug screening results obtained by Stukalov et al., 2021 [38]. First, we gathered all drug expression data from the LINCS L1000 dataset for the A549 cell line [39]. Next, we selected all drugs that were also tested in Stukalov et al. yielding 27 drugs [38]. For each drug we matched the concentration values from the L1000 datasets to Stukalov et al. building a dataset of 81 drug-concentration transcriptomic experiments. For each experiment, we gathered the DEGs and performed PHENSIM simulation as described in this manuscript to produce specific drug signatures. For the pathogen signatures we used the expression data from Blanco-Melo et al. for A549 cell lines [14]. Finally, using the repurposing procedure, we determined the correlation values for each drug experiment and computed the FDR-adjusted p-values. The results were then compared to the Stukalov et al. *in vitro* drug screening results. Stukalov et al. reported as treatment-induced changes in virus growth over time using the log2 fold change of green fluorescent protein (GFP) signal normalized to the total cell confluence between the treated and control conditions [38].

To perform this comparison, we selected our repurposing candidates (negatively correlated drugs) and the viable candidates as reported by Stukalov et al. We then calculated the intersection percentage between the results of the two methods (Perc). To determine the significance of the results, we used a permutation test. First, for each drug, we build N random signatures as described above. Next, we compute the Pearson correlation coefficient against the pathogen profile for each signature. Then, we determine repurposing candidates by selecting negatively correlated drugs and compute the percentage of candidates overlapping with Stukalov et al. results ($PercR_{i}$). Finally, we count the number of times we obtain a greater percentage as:

$$p=\frac{\left|\left\{i|PercR_{i}>Perc\right\}\right|}{N}$$

A significant p-value (p < 0.05) indicates that our results are not obtained by chance.
